# Integrated microoptical system for continuous fluorescence monitoring of microtissues

**DOI:** 10.1038/s41378-025-01073-4

**Published:** 2025-11-12

**Authors:** Xu Tian, Hanie Kavand, Martin Köhler, Jessika Jessika, Reison Gjaci, Montse Visa, Per-Olof Berggren, Göran Stemme, Wouter van der Wijngaart, Anna Herland, Niclas Roxhed

**Affiliations:** 1 Division of Micro and Nanosystems, Department of Intelligent Systems, School of Electrical Engineering and Computer Science, KTH Royal Institute of Technology, Stockholm, Sweden; 2 Division of Nanobiotechnology, Department of Protein Science, SciLifeLab, KTH Royal Institute of Technology, Solna, Sweden; 3 The Rolf Luft Research Center for Diabetes and Endocrinology, Karolinska Institutet, Solna, Sweden; 4 AIMES – Center for the Advancement of Integrated Medical and Engineering Sciences, Karolinska Institutet and KTH Royal Institute of Technology, Stockholm, Sweden; 5 Department of Neuroscience, Karolinska Institutet, Solna, Sweden; 6 MedTechLabs, Bioclinicum, Karolinska University Hospital, Solna, Sweden

**Keywords:** Electrical and electronic engineering, Optical sensors

## Abstract

Microphysiological systems (MPS) are advanced in vitro platforms engineered to replicate in vivo conditions for studying human biology, disease mechanisms, and drug responses with greater physiological relevance. Fluorescence sensing is widely used as a functional readout in MPS due to its high sensitivity, selectivity, and stability. However, conventional fluorescence sensing systems often rely on bulky instrumentation with limited integration, which restricts continuous in situ monitoring, scalable high-throughput analysis, and spatially resolved investigation in multi-organ-on-a-chip models. To address these limitations, we present a highly miniaturized, fully integrated optical system with a 1 mm² footprint, enabling continuous in situ fluorescence monitoring of three-dimensional microtissues in close proximity. The system integrates microscale illumination and sensing units for fluorescence excitation and selective detection, an optical element for guided light propagation, and a microcage for mechanical confinement of microtissues. To demonstrate its capabilities, we integrated the miniaturized optical system with an MPS-relevant platform to monitor fluorescence signals in transgenic mouse pancreatic islets expressing genetically encoded calcium indicators. The integrated platform enables real-time, continuous monitoring of islet responses to potassium chloride stimulation and tracking of calcium oscillations for over two hours, providing valuable information about the functional status of the pancreatic islets. Our work enhances the analytical capabilities of MPS through the integration of miniaturized on-chip quantitative assessment tools, enabling precise, in situ, and continuous monitoring of biological activities in close proximity.

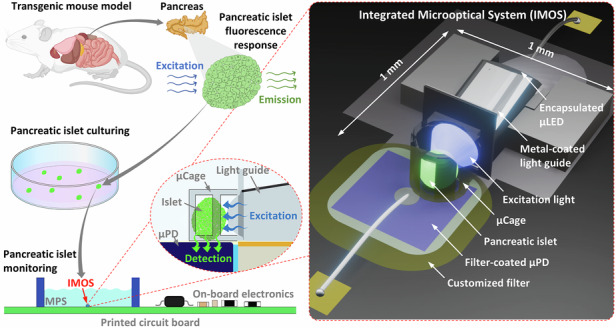

## Introduction

Understanding the functionality of cells, tissues, and organs ex vivo is crucial in many life science research fields, including drug screening, regenerative medicine, and procedures involving cell or organ transplantation^1–5^. Presently, monitoring methods in laboratory experiments and pre-clinical trials predominantly depend on benchtop analytical systems and animal models. These methods, however, are costly, often require large equipment, and raise ethical concerns^6–8^. To address these challenges, technologies known as “microphysiological systems” (MPS) or “Organs-on-Chips” (OoC) have been developed. These laboratory models combine biological elements with controlled physiological microenvironments on engineered chips to closely replicate in vivo conditions without the requirements of animal models or clinical trials^8,9^. The development of MPS leverages multiple sophisticated techniques, including microfabrication, fluidic control, extracellular matrix engineering, and microenvironmental control, to recreate complex biological models outside the body^9–11^. To enhance the physiological and pharmacological relevance of MPS, it is increasingly essential to integrate real-time, on-chip quantitative assessment tools. Such integration enables spatially and temporally resolved monitoring of cellular functions, drug responses, and microenvironmental parameters^10,11^. Therefore, a variety of detection methodologies have been integrated into MPS^12,13^. Among these, optical detection methods, such as surface plasmon resonance^14^, light absorption and quenching^15^, light scattering^16^, and fluorescence^17^ have been widely implemented due to their high sensitivity, robustness, non-destructive nature, broadband capabilities, and extensive application range^12^. Fluorescence detection, in particular, remains one of the most widely used sensing techniques in MPS due to its exceptional selectivity and stability^12,18^. It has been successfully applied in diverse MPS-related applications, including biomedical diagnostics^19^, environmental monitoring^20–22^, and cellular metabolite detections^23,24^. Despite their analytical strengths, the lack of miniaturization and integration of fluorescence excitation and detection modules typically results in relatively bulky instrumentation and an extended distance between biological interfaces and sensing elements. This limits their capacity for continuous in situ monitoring and hinders the development of compact MPS for cost-effectiveness and scalable high-throughput analysis^10,12^. While miniaturization and system-level integration present viable pathways to address these limitations, efforts to miniaturize and integrate fluorescence sensing systems in MPS-related applications have been constrained by the physical size of optoelectronic components and the fabrication of compact optical elements^25–28^. Moreover, long-term monitoring within fluidic chips often requires spatial confinement of cells, commonly achieved through hydrogels^29^. However, these materials can alter the physicochemical properties of the microenvironment and potentially affect cell function^30,31^. Together, these challenges restrict long-term, continuous in situ monitoring within MPS and limit the development of compact platforms for MPS-related applications. As a result, there is a growing need to develop downscaled, fully integrated on-chip fluorescence monitoring modules that can be seamlessly incorporated into MPS.

Here, we present a highly miniaturized integrated microoptical system (IMOS) for on-chip fluorescence monitoring of three-dimensional (3D) microtissues in close proximity. We selected pancreatic islets, spheroidal clusters of endocrine cells that regulate glucose homeostasis through hormone secretion, as an organoid-like microtissue model. Their complex cellular architecture, metabolic responsiveness, and disease relevance make them a physiologically relevant model for studying cellular signaling, particularly as a well-established model in the context of diabetes research^32,33^. By integrating the IMOS into an MPS-relevant platform, dynamic biological activities, including potassium chloride (KCl) stimulation and intracellular calcium (Ca²⁺) oscillations, were successfully detected and recorded in multiple mouse pancreatic islets. This work represents a key step toward the realization of fully integrated MPS equipped with miniaturized on-chip analytical tools for noninvasive, continuous in situ monitoring of microtissues. This advancement facilitates future development of MPS-based models with enhanced precision and analytical throughput.

## Results and discussion

### Design, simulation, and fabrication of the IMOS

**Fig. 1:**
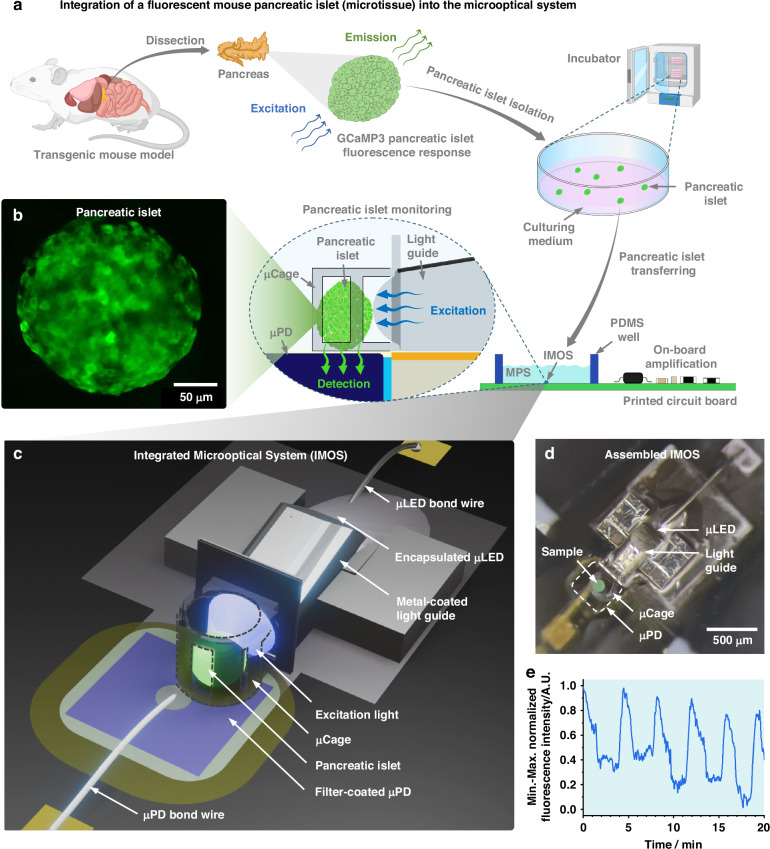
**An integrated microoptical system (IMOS) for continuous in situ fluorescence monitoring of a pancreatic islet from a transgenic mouse expressing the fluorescent biosensor protein GCaMP3.** **a** Schematic illustrating the isolation of a fluorescent pancreatic islet and its integration into the IMOS. **b** Confocal microscope image of an isolated GCaMP3-expressing mouse pancreatic islet. **c** 3D rendering of the IMOS, highlighting integrated optical components for close-proximity fluorescence detection of microtissues. **d** Photograph of the assembled IMOS containing a green fluorescent microbead (~150 µm in diameter) used for scale and visual demonstration. **e** Representative 20-minute recording of Ca²⁺ oscillations in a pancreatic islet, acquired using the IMOS

We designed our system based on pancreatic islets from a transgenic mouse expressing the engineered fluorescent Ca^2+^ biosensor protein GCaMP3 in their pancreatic β-cells (Fig. 1a)^32,34^. GCaMP3 is a genetically encoded calcium indicator, created by fusing green fluorescent protein (GFP) and the Ca^2+^-binding protein calmodulin^35^. Upon Ca^2+^ binding, calmodulin undergoes a conformational change that increases GFP fluorescence emission, reflecting changes and providing a readout of Ca^2+^ level fluctuations^35^. The GCaMP3 fluorophore exhibits a peak excitation wavelength at *λ* = 480 nm and a peak emission wavelength at *λ* = 510 nm. This enables us to study the natural and rhythmic fluctuations in intracellular Ca^2+^ concentrations (Ca^2+^ oscillations) that occur in the β-cells of the pancreatic islets by monitoring the emitted fluorescence over a period of time. The pancreatic islet from the transgenic mouse was isolated, cultured, and transferred to the IMOS housed within the MPS-relevant platform, enabling continuous in situ monitoring of dynamic islet activities through fluorescence signal variations with high selectivity and close proximity. An optical microscopic image of the isolated islets is shown in Fig. S1a. Figure 1b shows a confocal microscope image visualizing fluorescence from a typical GCaMP3-expressing transgenic mouse pancreatic islet.

Figure 1c presents an overview of the IMOS designed for localized fluorescence monitoring of pancreatic islets. An optical element (light guide) was engineered to effectively guide the light emitted by a micro light-emitting diode (µLED) to efficiently illuminate the pancreatic islet held within a microcage (µCage). A micro photodiode (µPD) is positioned beneath the µCage to selectively detect the fluorescence emitted by the islet. Figure 1d shows the fabricated IMOS with a footprint of ~1 mm^2^. A green fluorescent microbead with a diameter of approximately 150 µm was placed in the µCage as a size reference. The IMOS was fabricated using low-autofluorescence materials, resulting in minimal background fluorescence signal within the primary emission window of GCaMP3 (500–550 nm), as demonstrated by the fluorescence microscope image in Fig. S1b. By incorporating the IMOS into the MPS-relevant platform along with external electronic peripherals, dynamic and rhythmic intracellular Ca²⁺ oscillations were successfully detected and recorded, as illustrated in the representative results shown in Fig. 1e. Compared to β-cell Ca²⁺ dynamics obtained with conventional stage-based fluorescence microscopy^32,34^, which relies on bulky optics, separate excitation and detection modules, and manual alignment, the IMOS provides a comparable functional readout through a fully miniaturized and integrated design. Its small footprint allows straightforward placement inside MPS-relevant devices and facilitates scalability through device parallelization. In addition, the close proximity between the optical components and the islet could potentially improve signal collection efficiency and support stable, continuous in situ monitoring of islet activity, enabling more physiologically relevant assessments over extended periods. Furthermore, the compact form factor of the IMOS also raises the possibility of integrating the system together with an MPS-like chamber entirely inside a standard incubator. In this configuration, islets could be maintained under tightly controlled culture conditions while being continuously monitored without interruption. Such an approach would reduce experimental variability introduced by repeated transfers of islets between the incubator and monitoring platform, enabling the collection of long-term datasets capturing slow physiological changes or progressive responses to pharmacological agents. This capability is particularly valuable for studying chronic islet function, disease progression, and drug efficacy in a setting that more closely reflects the natural cellular environment.

**Fig. 2:**
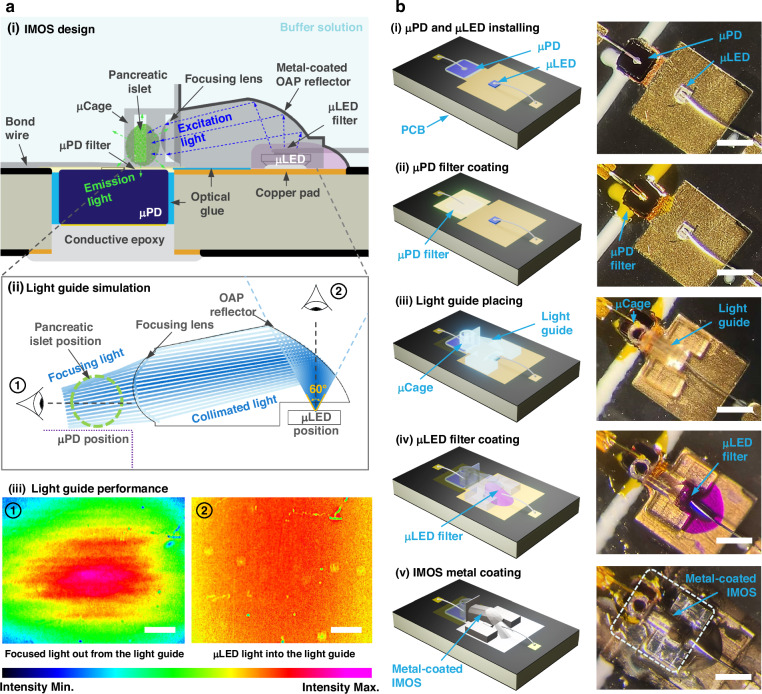
**Design and fabrication of the integrated microoptical system (IMOS).** **a** IMOS design: (i) Cross-sectional schematic of the IMOS. (ii) Ray-tracing simulation results of the designed light guide, performed using the ray optics module in COMSOL Multiphysics. (iii) Experimental evaluation of light guide performance using a 4 × 6 mm^2^ CCD sensor placed ~1 cm from the light sources along the viewing axis, comparing two scenarios: the focused light emitted from the light guide output (left) and the unfocused LED input measured without the light guide (right). Scale bars: 1 mm. **b** Fabrication steps: 3D schematic (left) and top-view photographs (right) illustrating the key fabrication steps of the IMOS. Scale bars: 500 µm

Figure 2a–i presents a cross-sectional schematic illustration of the light transmission path within the IMOS. The 3D-printed light guide comprises a cavity for housing the filter-coated µLED, an off-axis parabolic (OAP) reflector coated by a thin aluminum layer, and a focusing lens. Light from the µLED passes through a customized short-pass absorptive optical filter, propagates through the clear 3D-printed material, and is redirected by the metal-coated OAP reflector through the focusing lens to illuminate the pancreatic islet in the 3D-printed µCage. The µPD, coated with a thin long-pass absorptive optical filter, is positioned below the µCage to selectively detect the fluorescence emitted by the pancreatic islet. The compact light guide was designed using geometrical optics principles and simulated using the Ray Optics module of COMSOL Multiphysics software (Section S2–S4, Figs. S2–S4)^36,37^. As shown in the simplified two-dimensional (2D) ray-tracing simulation results (Fig. 2a–ii), the designed light guide redirects, collimates, and focuses light from a point source in the µLED, with an emission angle of up to 60°, onto the pancreatic islet in the µCage. The functionality of the light guide was verified using a light intensity spatial distribution analyzer in air (Fig. S5), and a similar light intensity distribution was expected in water (Fig. S3b). Figure 2a–iii shows relative light intensity spatial distributions, represented as pseudo-color images, at two positions of interest (light guide output and input) using identical sensor settings and gains. These measurement results indicate that the light guide successfully redirects the predominantly top-emitted light from the µLED, which follows a Lambertian radiation pattern, laterally toward the sample region, in agreement with the ray-tracing simulation results. This optical redirection enables the efficient illumination of the pancreatic islet, positioned within the µCage, by producing a focused output beam directed at the target location. A comparative analysis of conditions with (Fig. S6a) and without (Fig. S6b) the light guide further reveals that, in the absence of the guide, only a minimal fraction of the µLED-emitted light contributes to fluorescence excitation, as most of the light disperses away from the sample region. Together, these results confirm that the designed light guide is capable of effectively redirecting and focusing light for the efficient illumination of the pancreatic islet positioned in the µCage. Fabrication results for key steps in the IMOS assembly process are shown in Figs. 2b and S7.

### Characterization of custom absorptive optical filters and operating characteristics of µLED and µPD

**Fig. 3:**
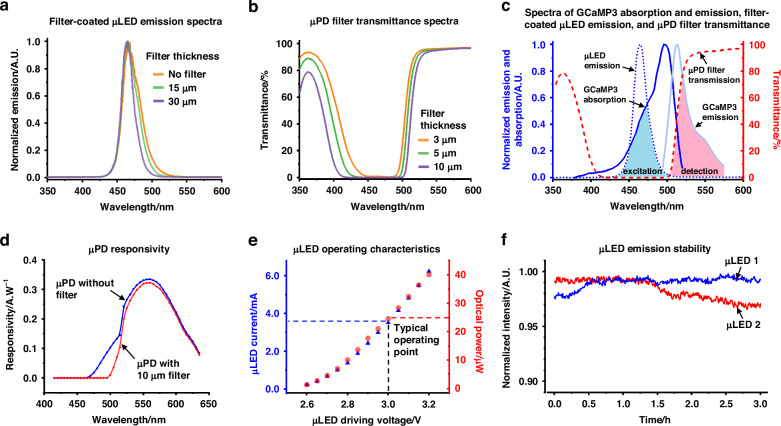
**Characterization of customized absorptive optical filters and operating characteristics of µPD and µLED.** **a** Emission spectra of µLEDs coated with filters of different thicknesses. **b** Transmission spectra of µPD filters at varying thicknesses. **c** Overview of GCaMP3 absorption and emission spectra, the emission spectrum of a µLED coated with a 30 µm-thick filter, and the transmission spectrum of a 10 µm µPD filter, demonstrating effective separation of excitation and detection wavelengths for background signal suppression in IMOS operation. **d** Responsivity of µPD without a filter and with a 10 µm-thick optical filter. **e** Driving current and emitted optical power of a typical µLED as a function of applied voltage during operation. **f** Emission stability of two typical µLEDs driven by an LED driver over a 3-hour operating period

To optimize the spectral performance of the excitation and the detection units for selective fluorescence detection from mouse pancreatic islets, we developed polymer-based short-pass and long-pass absorptive optical filters. The characterization results of these filters, along with the operating characteristics of the integrated optoelectronic components within the IMOS, are presented in Fig. 3. Dye-doped optical filters were fabricated using SU-8 as the base polymer, with an average transmittance of 96% between 450-490 nm (dominant excitation wavelengths) and 97% between 500 and 550 nm (fluorescence detection window), as shown in Fig. S8. A µLED with a peak emission wavelength of 470 nm was selected to maximize spectral overlap with the GCaMP3 absorption spectrum, thereby enhancing fluorescence excitation, while minimizing overlap with the GCaMP3 emission spectrum to reduce background signals. To further suppress spectral crosstalk between µLED emission and GCaMP3 fluorescence detection, we investigated narrowing the µLED emission profile through absorptive optical filter coatings. Figure 3a presents a comparative analysis of µLED emission spectra after coating with optical filters of varying thicknesses. A 30 µm-thick filter was found to effectively block wavelengths above 500 nm without significantly attenuating overall light output, whereas thinner filters were less effective in suppressing unwanted spectral components. In addition to optimizing the excitation source, we developed a long-pass absorptive filter for the µPD to achieve selective detection of fluorescence emitted from GCaMP3. Figure 3b illustrates the relationship between filter thickness and transmission spectrum for the µPD filter. A 10 µm-thick filter layer effectively blocks light from the µLED while allowing high transmission above 510 nm. In contrast, thinner thicknesses of 3 µm and 5 µm exhibit inadequate blocking due to increased transmission at shorter wavelengths (<500 nm). Although the 10 µm-thick filter may slightly obstruct signals from GCaMP3 due to low transmission ~500 nm wavelength, it was deemed optimal due to its improved spectral selectivity and consistent coating outcomes.

Figure 3c provides an overview of the filter-coated µLED emission spectrum, GCaMP3 excitation and emission spectra, and transmission profile of the optical filter applied to µPD. For IMOS operation, a 30 µm-thick absorptive filter was placed between the µLED and the light guide, resulting in a shifted peak emission at 465 nm and minimal intensity above 500 nm. GCaMP3 absorbs this filtered excitation light and emits fluorescence with a peak at 510 nm. A 10 µm-thick long-pass filter coated on the µPD blocks the excitation light while transmitting the emitted fluorescence signal. The shaded regions labeled ‘excitation’ and ‘detection’ in Fig. 3c highlight the spectral overlap between GCaMP3 absorption and filtered µLED emission, and between GCaMP3 emission and µPD filter transmission, respectively. This design ensures effective spectral separation between excitation and detection channels, enabling selective fluorescence detection while suppressing background interference. Since the entire IMOS was coated with a parylene-C layer for biocompatibility and encapsulation, we evaluated its effect on the optical properties of the integrated filters. Based on previous work^38^, our calculation indicates that a 10-µm-thick parylene-C layer exhibits ~90% transmittance in the 400–650 nm wavelength range (Fig. S8). The relatively low autofluorescence of parylene-C for our target application has also been confirmed by the fluorescence microscope image shown in Fig. S1b.

The operating characteristics of the optoelectronic devices within IMOS were further investigated. Figure 3d presents the responsivity spectra of a bare µPD die and a µPD coated with a 10 µm-thick absorptive optical filter, both operated in photovoltaic mode to minimize noise. The measurement results show that the filter effectively suppresses the µPD responsivity at wavelengths below 500 nm (corresponding to the µLED emission spectrum), while preserving high responsivity within the 500–550 nm range, which corresponds to the primary emission window of GCaMP3. These findings confirm that the optical filter enables selective rejection of excitation light while allowing efficient detection of the fluorescence signal. Under standard operating conditions, the µLED was driven at 3.0 V, drawing a current of 3.6 mA, and the IMOS delivers an optical power output of ~25 µW at the location of the pancreatic islet (Fig. 3e). This low illumination power reduces the risk of phototoxic effects during fluorescence monitoring^39^. To evaluate the stability of the µLED and its driving system, two µLEDs were repeatedly pulsed with a width of 180 µs and operated continuously in air at ambient temperature for 3 hours (Fig. 3f). The results indicate that the µLEDs, along with their driving circuitry, exhibit minor performance fluctuations over time, with relative power output variations remaining within a few percent.

### Pancreatic islet monitoring platform and electrical acquisition system

**Fig. 4:**
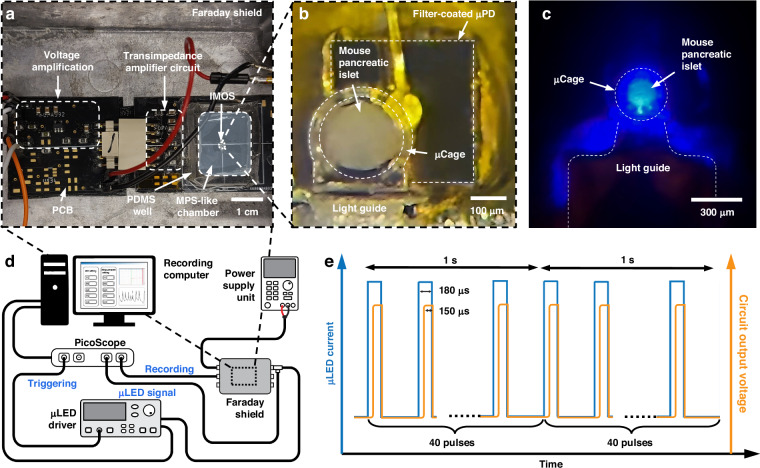
**Pancreatic islet monitoring platform and electrical acquisition system.** **a** Photograph of the pancreatic islet monitoring platform, featuring an MPS-like chamber and on-board electronics integrated on a single PCB that is enclosed within a Faraday shield. **b** Close-up photograph of a mouse pancreatic islet held by the µCage on top of a filter-coated µPD inside the microenvironment. **c** Image of a pancreatic islet during the monitoring process through the on-chip IMOS. **d** Schematic of the computer-controlled acquisition system. **e** Timing diagram of µLED excitation and µPD detection pulses driven by the computer program for fluorescence excitation and data acquisition

The platform developed for real-time, dynamic monitoring of pancreatic islets is shown in Fig. 4a. A customized printed circuit board (PCB) integrates the MPS-like chamber, which houses the IMOS, along with a circuit connecting the µLED to its driver and a two-stage amplification circuit (Fig. S9) used to convert and amplify the photocurrent generated by the µPD. An external LED driving system controls both the intensity and periodicity of the µLED emission. To validate the functionality of the fully integrated opto-electrical system, we compared the fluorescence signals detected from a fluorescent bead using the IMOS against those obtained with a simplified optical configuration without the light guide. As shown in Fig. S10, fluorescence signals detected by IMOS were significantly stronger than the simple configuration with only µLED and µPD, aligning with the earlier observations in Fig. 2a–iii. Figure 4b provides a close-up view of the IMOS detection region, showing a pancreatic islet positioned within the µCage inside the MPS-like chamber. This consists of a polydimethylsiloxane (PDMS) well filled with a temperature-controlled HEPES-buffered solution to maintain a physiological environment during experiments. To prepare pancreatic islets for study, the pancreas was dissected from a transgenic mouse, enzymatically digested to isolate individual islets, and cultured under standard incubator conditions to preserve viability and function^32^. A single islet was then transferred to the monitoring platform for experimentation. During operation, localized light emitted from the µLED illuminates the pancreatic islet positioned in the µCage via the light guide (Fig. 4c), allowing selective and close-proximity fluorescence monitoring. Temporally resolved fluorescence signals were then recorded using a computer-aided electrical acquisition system.

The electrical acquisition system developed to operate the IMOS and measure real-time fluorescence signals is shown in Fig. 4d. To minimize phototoxicity during measurements, a pulsed excitation and detection strategy was employed (Fig. 4e). The system was continuously tested under various conditions to evaluate background signal levels originating from the electrical acquisition system, the on-board µPD signal amplification circuit, and the µLED excitation system (Fig. S11a). The relative strength of these background signals was then compared to the actual biological fluorescence signal detected from the pancreatic islet (Fig. S11b). Over 200-second measurement periods, the background signals from the acquisition system and the on-board µPD signal amplification circuit remained insignificant, with peak-to-peak amplitudes under 4% of the normalization range. The background signal caused by the excitation light gradually decreased with continued operation. The combined background signal from all three systems (acquisition, on-board amplification, and µLED excitation) eventually stabilized at ~6% of the normalization range. Following the placement of a pancreatic islet into the IMOS µCage, the platform successfully detected distinct intracellular Ca²⁺ activity and its response to KCl stimulation (Fig. S11b). During a 720-second (12-minute) recording session, slow Ca²⁺ oscillations were observed as periodic waveforms with clear peak-to-peak variations, demonstrating successful monitoring of islet function. Upon addition of 25 mM KCl, a pronounced fluorescence peak corresponding to a Ca²⁺ influx through voltage-gated Ca²⁺ channels was detected, confirming the platform’s sensitivity to physiological stimulation.

### Dynamic monitoring of pancreatic islets

**Fig. 5:**
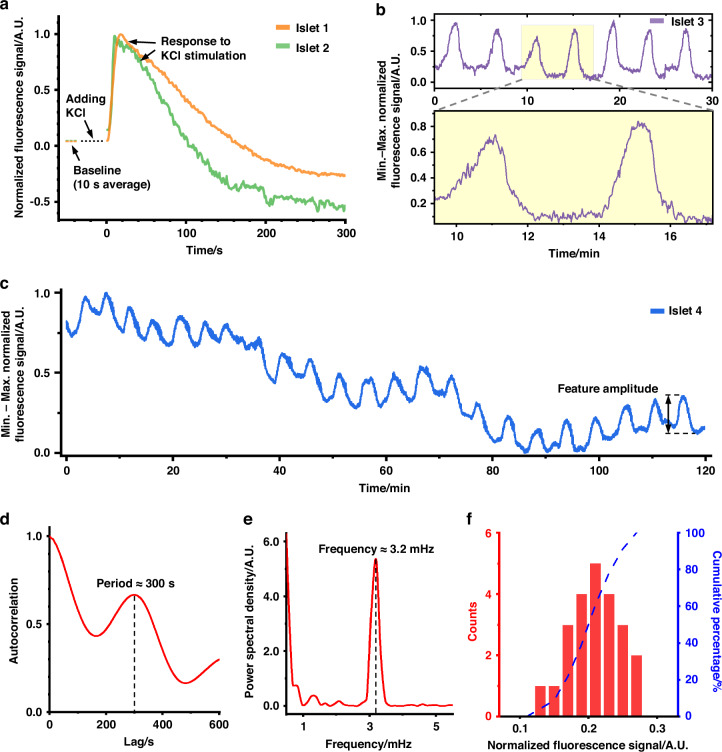
**Pancreatic islet activities recorded using the IMOS-integrated measurement platform and associated electronic peripherals.** **a** Responses to KCl stimulation recorded from two distinct islets (Islet 1 and Islet 2). The results were normalized between the baselines (the average value of 10-second readouts before adding KCl, signal = 0) and the maximum recorded values after KCl addition (signal = 1). **b** 30-minute activity trace of an islet (Islet 3) recorded using IMOS with a filter-coated 460 nm peak emission µLED. Data were normalized by min–max scaling over the recording interval. **c** 2-hour activity trace of an islet (Islet 4) recorded under the same conditions, normalized by min–max scaling. **d** Autocorrelation analysis of the Ca²⁺ oscillations in Fig. 5c. Peaks confirm the rhythmic nature of the oscillations and indicate a dominant oscillation period of ~300 s (~3.3 mHz). **e** Frequency-domain analysis of the oscillations in Fig. 5c using Fast Fourier Transform (FFT), showing a dominant frequency component at ~3.2 mHz. **f** Histogram of oscillation amplitudes from Fig. 5c, illustrating the distribution of Ca²⁺ response strengths and the heterogeneity of islet activity

Figure 5 presents the recorded activity of pancreatic islets using the IMOS-integrated monitoring platform. The transgenic pancreatic islet, previously described in Fig. 1a–i, serve as a biologically relevant in vitro and in vivo microtissue model characterized by well-known Ca²⁺ dynamics. These dynamics are monitored through GCaMP3 fluorescence intensity, which correlates with cytoplasmic free Ca²⁺ concentration. Under intermediate glucose conditions (e.g., 11 mM glucose), the islets exhibit oscillatory intracellular Ca²⁺ concentrations. Glucose metabolism leads to the closure of ATP-regulated K⁺ channels, causing membrane depolarization. This depolarization subsequently opens voltage-gated Ca²⁺ channels, resulting in increased intracellular Ca²⁺ levels and ultimately insulin secretion. Figure 5a shows the typical responses of two different islets (Islet 1 and Islet 2) to the addition of 25 mM KCl. KCl induces immediate membrane depolarization, triggering a rise in intracellular Ca²⁺ through the opening of voltage-gated Ca²⁺ channels. Upon KCl addition, fluorescence signals from both islets rapidly increased, peaking shortly thereafter, and gradually returned to baseline levels after ~105 seconds for Islet 1 and 170 seconds for Islet 2. Figure S12a displays a 30-minute segment of fluorescence signals from another two islets. The raw signals exhibited certain fluctuations due to considerable background noise and system-level interference, which were post-processed using a fast Fourier transform (FFT) filter. The filtered signals clearly revealed characteristic Ca²⁺ oscillations expected in functional islets.

To reduce background signals, the µLED in the IMOS was replaced with a variant featuring a peak emission wavelength of 460 nm, while retaining the overall design shown in Fig. 2a. A comparison of emission spectra for three different fluorescence excitation configurations is provided in Fig. S12b. Pairing the 460 nm μLED with the same 30 μm-thick absorptive optical filter resulted in a blue-shifted emission spectrum with a peak emission at ~458 nm. This modification further increased the spectral separation between the fluorescence excitation and the detection window of the filter-coated µPD, thereby reducing background interference. Figure 5b displays a 30-minute fluorescence recording of an islet (Islet 3) using the modified IMOS integrated with the 460 nm μLED. Compared to the recordings obtained using the 470 nm µLED configuration (Fig. S12a), a clearer and more regular Ca²⁺ oscillation pattern was evident, indicating enhanced signal quality and reduced background noise from the excitation unit due to improved spectral separation. We estimated the signal-to-noise ratios (SNR) for both scenarios by taking the ratio between the average root-mean-square (RMS) amplitudes of Ca²⁺ oscillation peaks and background signals over the recording periods (Fig. S13), the calculations show that the SNR for 470 nm µLED and 460 nm µLED configurations are 2.3 dB and 13.1 dB, respectively, further confirming a significantly suppressed noise floor through changing the µLED configuration. To validate repeatability of the recordings using the improved system, recordings of three additional islets using the same 460 nm µLED configuration were shown in Fig. S14a, b. Both figures exhibit well-defined, regular Ca²⁺ oscillation patterns. Additionally, monitoring of an islet (Islet 4) over a 2-hour period further demonstrated stable and consistent Ca²⁺ oscillation patterns (Fig. 5c). We performed autocorrelation and FFT on the 2-hour recording results shown in Fig. 5c to identify the periodicity and frequency of pancreatic islet Ca²⁺ oscillations; the results are presented in Fig. 5d and Fig. 5e, respectively. The autocorrelation quantifies the periodic nature of the Ca²⁺ oscillations by detecting repeating patterns in the recording over time, indicating a period of oscillation of ~300 s. The FFT process reveals that the dominant frequency of oscillation is ~3.2 mHz, closely aligned with the period value obtained through autocorrelation. We also conducted statistical analysis on the amplitude of Ca²⁺ oscillations within the 2-hour recording (Fig. 5f). Among the 23 peaks detected, most of the peaks have feature amplitudes ~0.2 of the full normalization range (min–max normalization). The mean and standard deviation were calculated to be 0.206 and 0.036, respectively. Lastly, we also noticed that the measurement results obtained from different islets show expected variations in signal strength based on factors such as their physical parameters (size and β-cell population), GCaMP3 expression levels, and centroid alignment between the islet and the optical axis of IMOS (Fig. S14c). The pancreatic islet, acting as a 3D scattering and fluorescent source, interacts with the focused excitation light spot. Detection accuracy depends strongly on islet size, biological parameters, and alignment between the centroid of the spheroid and the optical axis. Optimal performance occurs when a healthy islet that has a spheroid size well matched to the focused light spot at the sample plane and its centroid is aligned with the optical axis of the incoming excitation light. If the spheroid is smaller or if it has a lateral displacement, it leads to partial excitation of the islet, reducing total fluorescence yield and leading to signal distortion. If the spheroid is larger, excitation illuminates only partially, leading to under-sampling of the entire fluorescent spheroid and potential underestimation of functional activity.

Together, these results demonstrate the capability of IMOS for repeatable and continuous in situ monitoring of pancreatic islet Ca²⁺ oscillations. This functionality is crucial for understanding insulin secretion mechanisms and assessing β-cell functions, as these oscillations are tightly linked to regulated insulin secretion dynamics. Specific parameters of these signals, including amplitude, frequency, and regularity, carry important physiological meaning. The amplitude of oscillations reflects the strength of β-cell depolarization and subsequent Ca²⁺ influx, while frequency and rhythmicity are associated with the coordination of intracellular signaling throughout the pancreatic islet and the maintenance of pulsatile insulin secretion. Disruptions such as reduced amplitude, irregular oscillation frequency, or loss of synchrony between cells often indicate impaired β-cell responsiveness and are characteristic of type 2 diabetes^40^. By enabling precise, long-term measurements of these parameters under controlled conditions, the IMOS provides a powerful platform for probing islet physiology in health and disease. Beyond fundamental studies for investigating diabetic pathophysiology, its capabilities also hold considerable promise for drug testing and evaluating therapeutic interventions using MPS, as pharmacological agents that modulate Ca²⁺ oscillatory behavior can be evaluated directly within physiologically relevant microenvironments.

Importantly, this work advances the field of integrated and real-time optical sensing for complex in vitro models by enabling high spatial and temporal resolution measurements relevant to islet biology. The miniaturization and integration of the IMOS directly onto the MPS-relevant platform not only reduces the distance between the sensing elements and biological interfaces, potentially improving signal fidelity^10^, but also opens the door to scalable and multiplexed sensing architectures. The versatility of the platform also highlights its potential for broader applications, including monitoring microtissues from cardiac and neural systems. These features allow for the simultaneous monitoring of multiple microtissues or organoid types within a single platform, supporting parallel analysis of biological responses under varying conditions. Practically, the IMOS is comparable in size to the microtissue it monitors, falling within the same order of magnitude. This compact footprint and modular design facilitate seamless integration into multi-organ-on-a-chip models, enabling spatially resolved, organ-specific sensing within interconnected tissue networks^41^. This capability is particularly valuable for studying inter-organ communication, systemic drug effects, and complex disease models.

## Conclusions

In conclusion, we developed an IMOS for continuous in situ fluorescence monitoring of microtissues in close proximity. The IMOS incorporates a sub-millimeter light guide that redirects and focuses light from a µLED, equipped with a customized short-pass optical filter, onto a µCage that holds the microscale sample in a physiologically relevant environment. Fluorescence emitted by the microtissue is detected by a µPD integrated with a tailored long-pass optical filter. The compact design of the IMOS results in a total excitation and sensing area of ~1 mm², making the system significantly smaller than conventional alternatives and highly suitable for applications that require scalability. To validate the IMOS’s functionality, we used mouse pancreatic islets expressing the GCaMP3 Ca²⁺ biosensor in their β-cells as a representative microtissue model. We successfully monitored islet activities such as intracellular Ca²⁺ oscillations and responses to KCl stimulation, demonstrating the IMOS’s ability to resolve biologically relevant functional dynamics. By enhancing spectral separation between excitation and detection wavelengths, the platform enabled continuous recording of clear, regular, and stable Ca²⁺ oscillations over a two-hour period. As Ca²⁺ dynamics are indicative of pancreatic β-cell function and reflect an intact stimulus-secretion coupling mechanism for insulin release, these results highlight the system’s relevance for functional assessment of endocrine microtissues. Overall, our work demonstrates the potential of the IMOS to advance biomedical research by enabling the integration of MPS with miniaturized on-chip analytical tools. This advancement facilitates the development of next-generation in vitro platforms capable of continuous in situ monitoring of cellular, tissue, and organ dynamics with high precision and scalability. These capabilities pave the way for deeper insights into complex biological processes and intercellular interactions, with broad implications for disease modeling, therapeutic development, and regenerative medicine.

## Materials and methods

### Preparation of absorptive optical filters for µPD and µLED

Dye-doped optical filters, customized separately for the µPD and µLED, were prepared by mixing absorbing dyes into SU-8 photoresist (SU-8-2005, MicroChem, USA) at a 3.5% concentration. To suppress light at 470 nm (the µLED’s peak emission wavelength), an absorbing dye (FDB-006, Yamada Chemicals, Japan) with maximum absorption at 473 nm was used for the µPD filter. To further minimize spectral overlap between the µLED emission and the filter-coated µPD absorption, the µLED emission spectrum was modified using a blend of two absorbing dyes, FDG-007 and FDG-003 (Yamada Chemicals, Japan), in a 9:1 ratio, respectively. The dyes were dissolved in cyclopentanone (Sigma-Aldrich, USA), a solvent compatible with both SU-8 and the dyes, to ensure uniform distribution within the SU-8 polymer. Since the spectral characteristics and peak absorption of the dyes used for filter fabrication interfere with the photo-polymerizing spectrum of SU-8, the SU-8 mixture was thermally cured at 65 °C for 60 minutes.

### Characterizations of optical filters

To determine the optimum filter thickness for the µPD, dye-doped polymer films of varying thicknesses were spin-coated on glass slides (631-1550, VWR, USA), and their transmission spectra were measured on a UV/Vis spectrophotometer (UV2550, Shimadzu, Japan). Layer thickness was measured using a mechanical profilometer (DektakXT, Bruker, USA). To determine the optimum filter thickness for the µLED, films of various thicknesses were spin-coated on glass slides. The emission spectra of µLEDs with various filter thicknesses were measured by placing the coated glass slides in front of the µLED and analyzing the transmitted light using a fiber-coupled spectrometer (USB2000, Ocean Optics, USA). The optimum layer thickness was then measured using the mechanical profilometer.

### Fabrication of the optical element (light guide)

The light guide was fabricated using a high-resolution 3D printer (Photonic Professional GT2 system, Nanoscribe, Germany) and low-fluorescence photoresin (IP-Visio, Nanoscribe, Germany). The structure was printed on an indium tin oxide (ITO)-coated glass substrate without any prior surface treatments. Post-printing development was performed by immersing the glass substrate (with the polymerized resin facing sideways) in propylene glycol methyl ether acetate (PGMEA, Sigma-Aldrich, USA) for 2 hours, followed by a washing step in isopropyl alcohol (VWR, USA) for 5 minutes. After drying at room temperature, the structures underwent UV exposure at 45 mW cm^−2^ with a 365 nm wavelength for 3 minutes to ensure full crosslinking. Structures were then immersed in a drop of isopropanol alcohol and carefully detached using a high-precision tweezer.

### Assembling process of IMOS

A 600 µm-thick customized PCB (FR-4, JDB Technology, China) with a black solder mask was milled with a 480 × 480 µm^2^ square hole for accommodating the placement of the µPD (LA-PD16AP1, Light Avenue, Germany) with dimensions of 400 × 400 × 220 µm^3^. The µPD was fixed in place using a low-fluorescence optical adhesive (NOA63, Norland, USA). A µLED (C470UT190/C460UT190, Cree, USA) with dimensions of 190 × 190 × 50 µm^3^ was mounted on the PCB. Electrical connections for both µPD and µLED were made using conductive epoxy (CW2460, Circuit Works, USA), and wire bonding (4523D, Kulicke & Soffa, Singapore). The customized optical filter was manually applied to the µPD using a fine brush (size 0000, Winsor & Newton, UK) with a sub-mm tip size, and cured at 65 °C for 60 minutes in an oven (UN30, Memmert, Germany). The µPD filter thickness was measured using a mechanical profilometer, showing a typical value of 11 µm (Fig. S7a). The 3D-printed light guide was then mounted onto a 1.2 × 1.2 mm^2^ gold-coated copper trace on the PCB using the low-fluorescence optical adhesive. The alignment among the different components has been further discussed in Section S4. A filter solution was applied through capillary filling into a designed 30 µm gap between the µLED and the light guide (Fig. S7b), followed by oven curing at 65 °C for 60 minutes. Next, a 2 µm-thick layer of parylene-C (Galentis S.r.l., Italy) was deposited using a parylene coater (PDS 2010, SCS, USA) for electrical isolation. A sacrificial photoresist mask (SPR700-1.2, Microresist, Germany) was manually applied to cover the µCage, focusing lens, and the µPD. Then, a 500-nm-thick aluminum layer was deposited on the IMOS by sputtering (844NT, KDF, USA), followed by a lift-off process to create a reflecting coating that covered only the light-guiding section of the light guide. Finally, the system was coated with an 8 µm-thick parylene-C layer to ensure biocompatibility and provide hermetic encapsulation for protection from the physiological environment. A green fluorescent bead (UVOMS-BG-1.025 125–150 µm, Cospheric LLC, USA) that has a diameter within the range of ~125–150 µm was placed in the fabricated IMOS as a demonstrator.

### Measurement of light intensity spatial distributions

The functionality of the light guide was tested in air using a light intensity spatial distribution analyzer (LH-360, Shenzhen Lianhuicheng Technology, China). Light distributions emitted from the light sources under four different situations, shown in Fig. 2a–iii and Figure S6, were captured by the charge-coupled device sensor (4 × 6 mm^2^, 1028 × 600 pixels) of the analyzer positioned ~1 cm away (Fig. S5). The sensor is capable of detecting light wavelength ranges from 190 nm to 1100 nm, covering the full emission spectrum of the µLEDs. Throughout the experiments, the μLEDs were powered by a 3 V forward voltage, and the emitted light intensities were evenly attenuated to 0.25% of the initial intensity using an optical attenuator accessory to match the detection range of the sensor.

### Measurement of operating characteristics of optoelectronic devices

To obtain the responsivities of µPDs, the incident light power on the µPDs was measured using a collimated monochromatic light source consisting of a monochromator (SpectraMaster, Life Science Resources, UK) with a light guide and a collimating lens (Thorlabs, USA), and an optical power and energy meter (PM100USB with detector S170C, Thorlabs, USA). The photocurrent generated by the µPD at each wavelength was measured using an amplifier circuit, with the voltage output of a trans-impedance amplification circuit measured using a multimeter (3440, PeakTech, Germany). The voltage-current curve of µLED was measured with the multimeter (3440, PeakTech, Germany) together with a current-sensing resistor. The voltage-optical power curve of µLED was measured with the multimeter (3440, PeakTech, Germany) together with the optical power and energy meter (PM100USB with detector S170C, Thorlabs, USA). To measure the long-term stability of the µLED light emissions, the μLEDs were driven by a square wave generated through a frequency generator (5000 Series, PicoScope, UK) and an LED driver (LDP-3840B, ILX Lightware, USA), with an amplitude of 3 V, a period of 360 µs, and a duty cycle of 50%. The emitted light from the µLEDs was detected using a photomultiplier tube (R3896, Hamamatsu Photonics, Japan) and recorded using an oscilloscope (5000 Series, PicoScope, UK).

### Platform for dynamic monitoring of pancreatic islets

The customized PCB integrates the MPS-like chamber housing the IMOS, a circuit connecting the µLED to its driver, and a two-stage amplification circuit to convert and amplify the photocurrent generated by the µPD. The µPD was configured in a photovoltaic mode to minimize dark current, and its signal was amplified with a total gain of 1.1 × 10^7^ before being routed to an external acquisition system. To ensure accurate measurements, the platform was enclosed in an aluminum Faraday shield throughout the experiments, providing both background light blocking and electromagnetic shielding. A PDMS well with dimensions of ~30 × 20 × 5 mm^3^ was used to create a physiological microenvironment on the measurement platform. The well was filled with a HEPES-buffered solution (125 mM NaCl, 5.9 mM KCl, 2.56 mM CaCl_2_, 1.2 mM MgCl_2_, 25 mM HEPES, 11 mM glucose, and 0.1% BSA [pH 7.4]), providing a good physiological environment for the pancreatic islet during the experiment. An external heater was placed under the MPS-like chamber to maintain physiological temperature to ensure proper cell metabolic function.

### Isolation, culture, and transfer of pancreatic islets

Pancreatic islets were isolated from adult double heterozygous Ins1Cre:GCaMP3 male mice aged 8–10 weeks. Ins1Cre:GCaMP3 mice were generated by breeding of B6.Cg-*Ins1*^*tm1.1(cre)Thor*^/J and B6.Cg-*Gt(ROSA)26Sor*^*tm38(CAG-GCaMP3)Hze*^/J, both obtained from Jackson Laboratories. Ins1Cre:GCaMP3 mice were kept on C56BL/6 J background and backcrossed at the animal core facility at Karolinska Institutet. Littermates were housed in groups of five animals per cage on a 12 h light–dark cycle with ad libitum access to food. In brief, mice were sacrificed via cervical dislocation, followed by an incision in the abdominal region to access the internal cavity. To prevent the spread of the digestive buffer within the pancreas after the bile duct injection, the common bile duct was carefully clamped. The digestion buffer consisted of ice-cold collagenase P (1.5 mg mL^−1^, Roche, Switzerland) in washing buffer (Hanks’ Balanced Salt Solution (Gibco™, Thermo Fisher Scientific, USA) supplemented with BSA (2.5 mg mL^−1^, Fisher Scientific, USA) in HEPES (25 mM, Gibco™, Thermo Fisher Scientific, USA), and pH adjusted to 7.4). After perfusing the pancreas with digestion buffer, it was excised, placed in a vial containing washing buffer, and incubated in a 37 °C water bath for 25 minutes. The digestion solution was passed 22 through an 18 G steel needle and washed twice for 5 mins each. Islets were carefully handpicked under a stereo microscope and cultured under standard conditions (37 °C, 5% CO_2_, humidified air) in RPMI-1640 medium (Gibco™, Thermo Fisher Scientific, USA) supplemented with 10% FBS (Gibco™, Thermo Fisher Scientific, USA), L-Glutamine (2 mM, Gibco™, Thermo Fisher Scientific, USA), penicillin (100 IU mL^−1^, Gibco™, Thermo Fisher Scientific, USA), and streptomycin (100 μg mL^−1^, Gibco™, Thermo Fisher Scientific, USA) until further use. All experimental protocols adhered strictly to the guidelines for care and use of animals in research at Karolinska Institute and were approved by the Regional Animal Ethics Committee (Ethical permit: 17431-2021). To prepare the measurement platform, the MPS-like chamber was first wetted with ethanol to prevent air bubble entrapment within the structural voids, followed by gradual replacement with pre-warmed (37 °C) culture medium. A Petri dish containing isolated pancreatic islets was then taken out of the incubator, and individual islets were carefully handpicked under a stereo microscope. Using a hand pipette with tips suited for volumes <100 µL, the islets were gently transferred and positioned within the chamber structure. After placement, the complete pancreatic islet monitoring platform was moved to the Faraday shield, positioned on top of the external heater, and connected to the acquisition system for fluorescence monitoring.

### Acquisition system for dynamic monitoring of pancreatic islets

A computer running a LabVIEW program is interfaced with the hardware via USB connections. A frequency generator (5000 Series, PicoScope, UK) sent trigger pulses to the LED driver (LDP-3840B, ILX Lightware, USA), initiating pulses that drove the μLED. The pulse amplitude and width were controlled by the LabVIEW program. An oscilloscope (5000 Series, PicoScope, UK) monitored the μLED current via the LED driver and recorded the signal from the IMOS at the output of the on-board amplification circuit. The amplification circuit was powered by an external power supply unit (320-KA3005D, RND, Switzerland). The PCB, which integrated both the on-board circuits and the MPS-like chamber containing the IMOS, was housed within a Faraday shield. The μLED was driven at 40 pulses per second with a pulse width of 180 µs. To generate each representative 1-second measurement value, the first 20 pulses out of the 40 pulses were used. Specifically, the stable section between the rising and falling edges of each pulse was integrated, and the resulting values were averaged over 20 consecutive pulses to produce a single output voltage value every second. The entire system (including IMOS, monitoring platform, and acquisition system altogether) continuously operated for ~60 minutes every time before the recording (warm-up process), thereby achieving improved system stability and recording quality.

### Data presentation

The spectra of μPD filter and μLED emission presented in Figs. 3c and S12b were smoothed using Spline connections based on measurement data to reduce jaggedness for a better visualization. The measurement results in Fig. S11 were normalized using the sum of the background signals from the µPD amplification circuit and the acquisition system as the zero-signal baseline, and the peak response of the islet to KCl stimulation as the maximum (normalized signal = 1). All plots were recorded in the same run and displayed on a consistent 20% scale of the full normalized range for a straightforward and fair comparison. The results in Fig. 5a were normalized between the baselines (the average value of 10 seconds of readouts before adding KCl, signal = 0) and the maximum recorded values after the addition of KCl (normalized signal = 1). The data presented in Fig. S12a were post-processed using an FFT filter to enhance the clarity of islet activity. The Fourier transform was first applied to analyze the frequency components of the recorded signal, followed by low-pass filtering with a cutoff frequency of 0.16 Hz. The results shown in Fig. 5b–e were normalized using feature scaling with Min–Max normalization over the recording interval.

### Signal preprocessing

Fluorescence traces were imported into Python. The time axis was resampled to a uniform grid using the median sampling interval (Δ*t*, sampling rate *fs* = 1/Δ*t*). Each trace was linearly detrended to remove offsets and slow baseline drifts. *Filtering*: to separate oscillatory regimes, signals were filtered with a 4th-order zero-phase Butterworth filter using a 0.01 Hz cutoff (≈100 s period). This yielded a high-pass component (fast oscillations, <100 s) and a low-pass component (slow oscillations, >100 s). *Autocorrelation*: normalized autocorrelation functions (ACFs) were computed as the biased correlation of each signal with itself, divided by the zero-lag value. This produced unitless ACFs bounded between –1 and 1. Dominant periods were estimated from the first non-trivial ACF peak. *Fourier analysis*: frequency spectra were obtained with the FFT, applied to detrended signals windowed with a Hann taper. The single-sided magnitude spectrum ∣X(f)∣ and squared magnitude (power) were computed. Frequency axes were expressed in Hz and mHz, and converted to oscillation periods (*P* = 1/*f*) for interpretation. *Signal-to-noise ratio (SNR)*: to quantify relative oscillation strength, the RMS of the high-pass and low-pass filtered signals was calculated in Excel. The linear SNR was defined as the ratio of the RMS values (slow/fast). The decibel SNR was then obtained as SNR dB = 20*log10 (SNR linear). *Amplitude analysis*: the feature amplitude for each peak was obtained by taking the difference between the maximum and minimum of each peak using an image processing program, ImageJ. The histogram was then generated along with the calculation of the mean and standard deviation.

## Supplementary information

Supporting Information
