# Supplementary material for: Integrated microoptical system for continuous fluorescence monitoring of microtissues
Source: Microsyst Nanoeng. 2025 Nov 12;11:213. doi: 10.1038/s41378-025-01073-4 (PMC12612206; doi:10.1038/s41378-025-01073-4)
Supplement: Supplementary file 1 — Supporting Information [file 41378_2025_1073_MOESM1_ESM.docx]

Supplementary Information

Integrated Microoptical System for Continuous Fluorescence Monitoring of Microtissues

*Xu Tian ^1^†, Hanie Kavand ^1,2,^†, Martin Köhler ^3^†, Jessika Jessika ^2^, Reison Gjaci ^1^, Montse Visa^3^, Per-Olof Berggren ^3^, Göran Stemme ^1^, Wouter van der Wijngaart ^1^, Anna Herland ^2^*, and Niclas Roxhed ^1,4^**


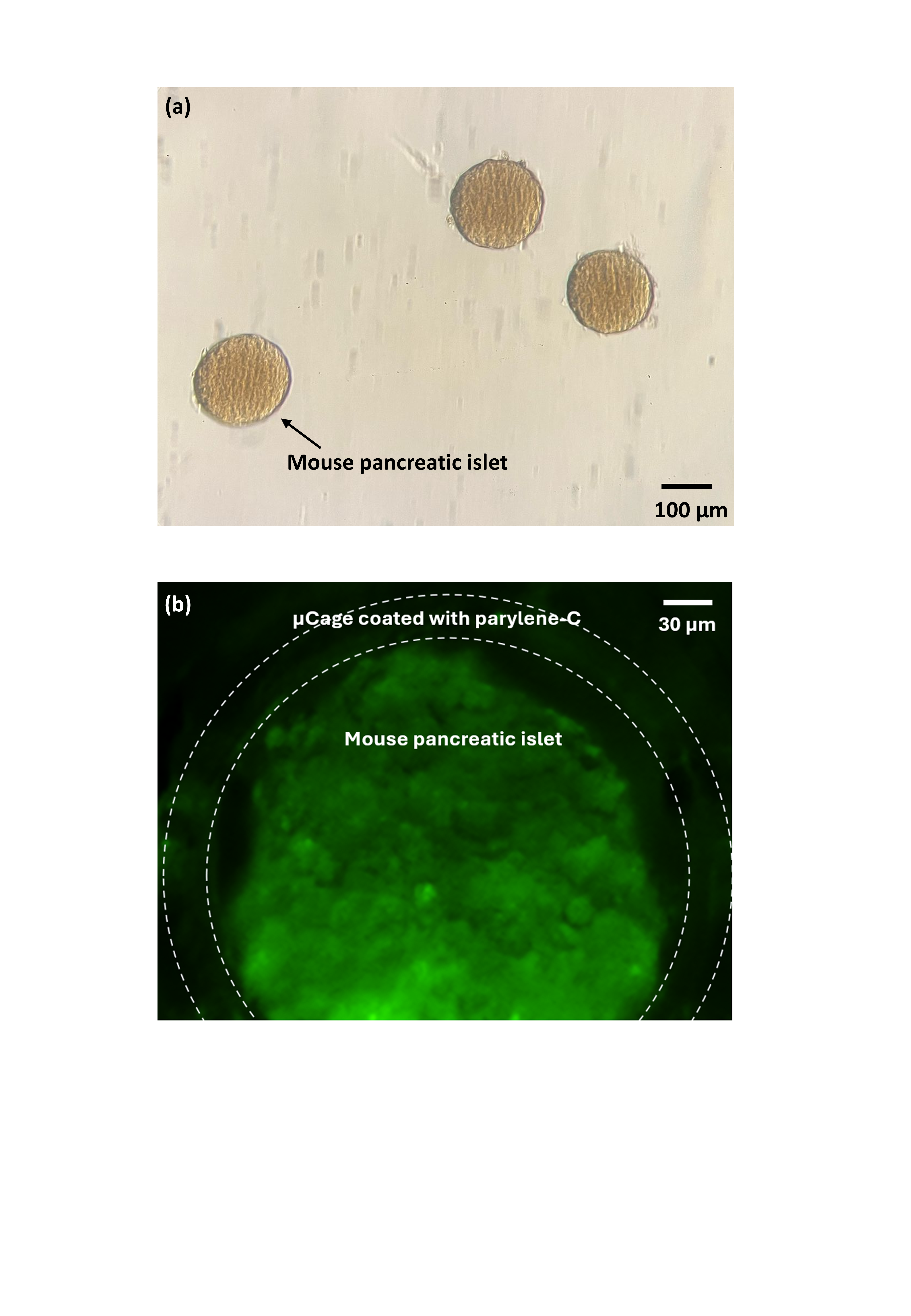


**Figure S1.** (a) Optical microscopic image of the isolated pancreatic islets. (b) Image of the fluorescence emission from a typical islet encoding GCaMP3 located in the µCage, captured using a fluorescence microscope. Compared to the fluorescence emitted by the islet (green color), much lower fluorescence emission from the µCage material coated with 10 μm parylene-C (which is the same as the light guide) and the surrounding environment was observed.


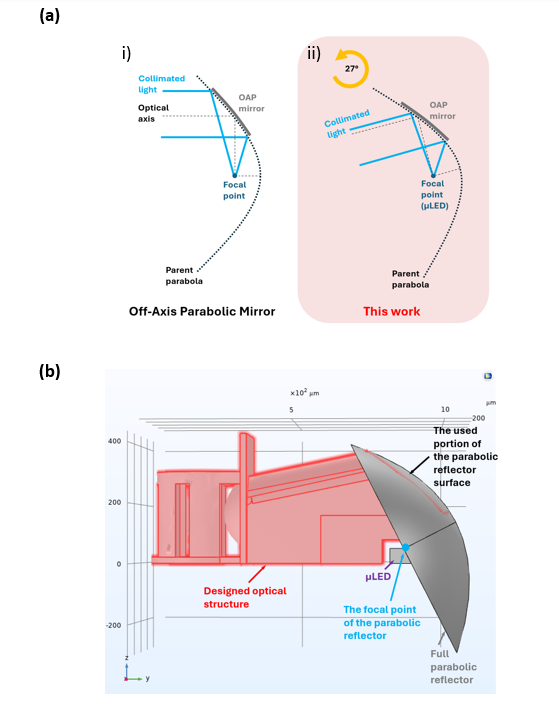


**Figure S2.** (a) Illustrations of the design of the OAP reflector: (i) Concept of full OAP reflector (ii) Rotation of OAP anticlockwise with an angle of 27° with respect to its focal point to meet the design requirements of this work. (b) 3D model of the OAP reflecting surface created using COMSOL Multiphysics based on ray-tracing simulation results.

**Section S2.** The compact light guide was designed using geometrical optics principles and simulated using the Ray Optics module of COMSOL Multiphysics software (version 6.0). The OAP reflector was designed based on a portion of a full parabolic reflector, which was generally used for taking light from a point source located at the focal point and creating a collimated beam (Figure S2-a). The 3D model of the OAP was built accordingly in COMSOL and exported for fabrication (Figure S2-b). The rim angle, center hole diameter, and focal length of the parabolic reflector shell designed for OAP are 90°, 0 µm, and 200 µm, respectively.


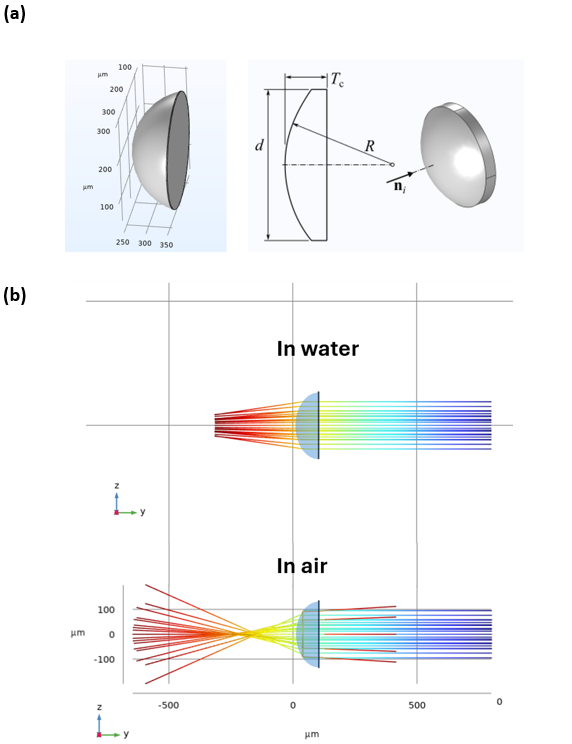


**Figure S3.** (a) Parameters of the focusing lens. (b) Ray-tracing simulation results of the standalone focusing lens in water and air.

**Section S3.** The focusing lens was designed based on a plano-convex lens with one spherical surface and one flat surface. The radius of curvature, center thickness, and lens diameter of the designed plano-convex lens are 140 µm, 105 µm, and 270 µm, respectively. After traveling through the light transmission channel, the collimated parallel light rays from the OAP reflector were converged by the plano-convex lens in both air and water, eventually focused on a single point.


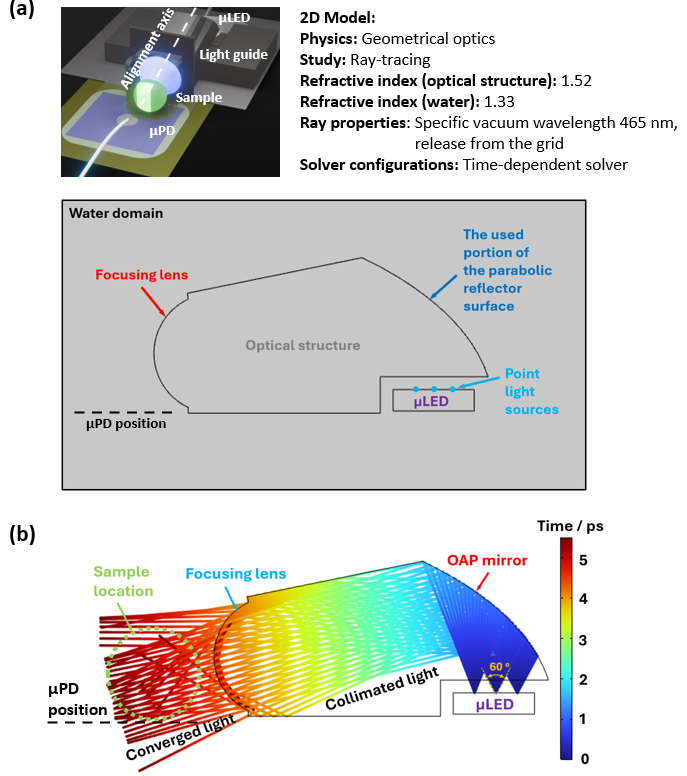


**Figure S4.** (a) 2D simulation model and parameters used in COMSOL for the designed light guide. (b) Ray-tracing simulation results with 3 light point sources at the top surface of µLED.

**Section S4.** For optimal fluorescence excitation and detection, the µLED, light guide, sample, and µPD should be aligned in the axial direction (Figure S4a). Since we designed the light guide to use the parabolic reflector to collimate a point source at the focus into a parallel beam, the µLED is expected to be positioned at or very close to the focal point of the parabolic reflector (Figure S2a). After travelling through the clear light guide structure, the collimated light was designed to be focused on the sample. The alignment includes centering the collimated beam on the focusing lens optical axis through its mechanical center (Figure S3b). The pancreatic islet (sample) is expected to be aligned with the converged light beam through the confinement of the μCage. Lastly, since the µPD has an active area of 400 x 400 μm^2^, which is much greater than the diameters μCage and pancreatic islet. Approximately a quarter of the µPD active area was positioned below the μCage and pancreatic islet and aligned with them to achieve the best optical performance.


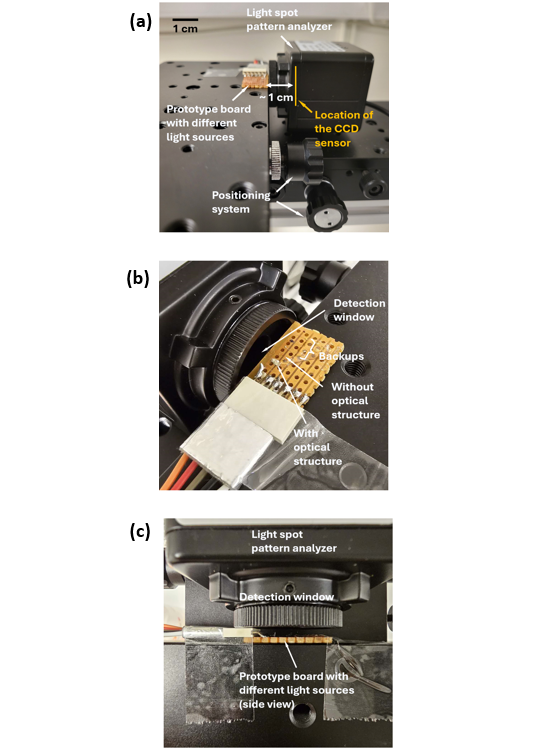


**Figure S5.** Light intensity spatial distribution measurement setup. (a) Setup overview. (b) Measurement setup for viewing the light distribution from the side. (c) Measurement setup for viewing the light distribution from the top.


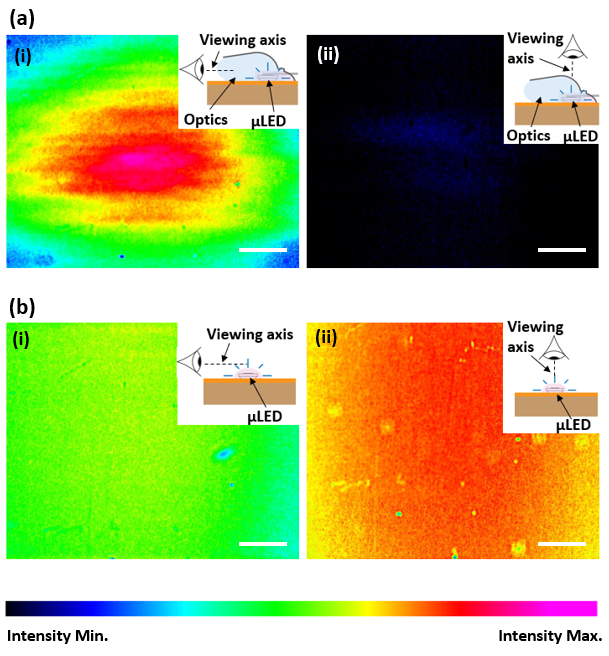


**Figure S6.** Comparison of light intensity spatial distributions, represented as Pseudo-Color images, between the scenarios (a) with and (b) without the light guide installed. Light intensity measurements were taken at four positions of interest: the side of the light guide (Figure S6a-i), the top of the light guide (Figure S6a-ii), the side (Figure S6b-i), and the top (Figure S6b-ii) of the filter-coated μLED. Measurements at the side and top (Figure S6a) of the light guide demonstrate that IMOS could effectively guide the light emitted by the μLED through the focusing lens for fluorescence excitation, consistent with the simulation results. In contrast, without the light guide (Figure S6b), only a small fraction of light emitted from the side of the μLED can be utilized for fluorescence excitation as most light emitted from the μLED top surface does not illuminate the sample region.


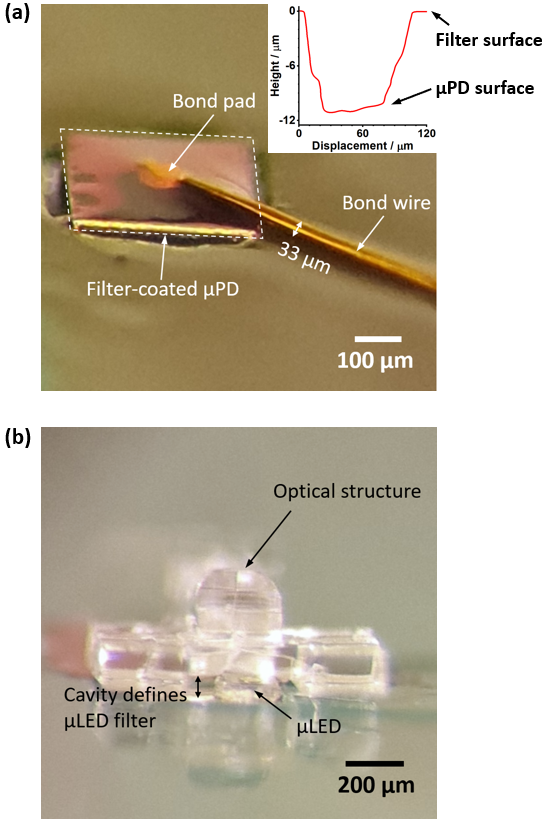


**Figure S7.** Thicknesses control of µPD and µLED filters: (a) Control and estimation of µPD filter. (b) Control and estimation of µLED filter.


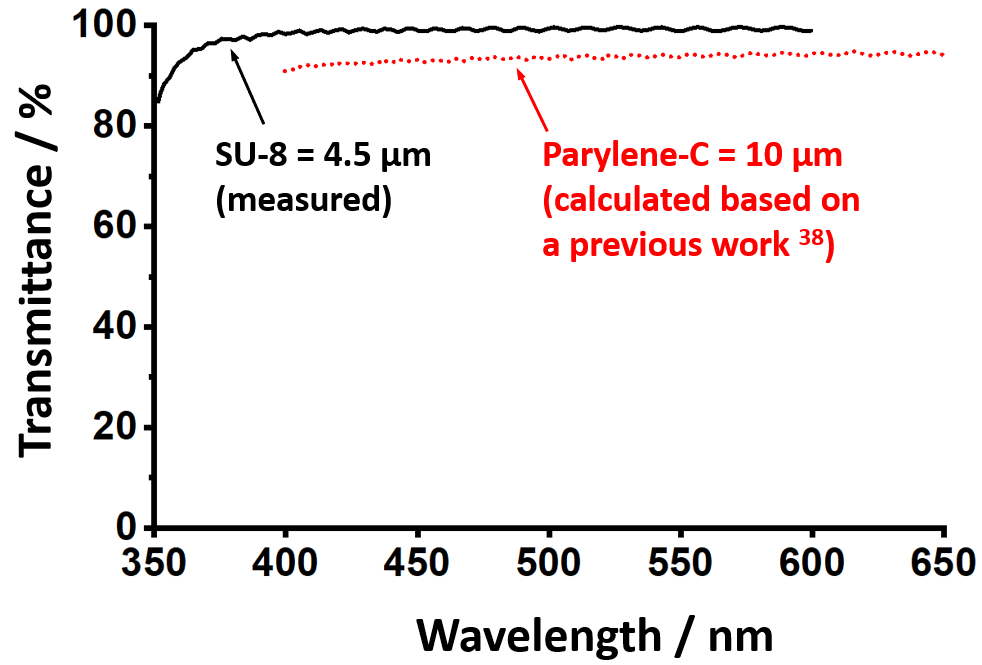


**Figure S8.** The transmittance spectra of SU-8 as the base polymer of the dye filter (thickness of 4.5 µm), measured using a spectrophotometer (UV2550, Shimadzu, Japan), and a 10-μm-thick parylene-C as an encapsulation layer to provide biocompatibility and protection from the physiological environment (calculated based on a previous work ^38^).

**Figure S9.** Electrical circuit diagram for operating the IMOS.


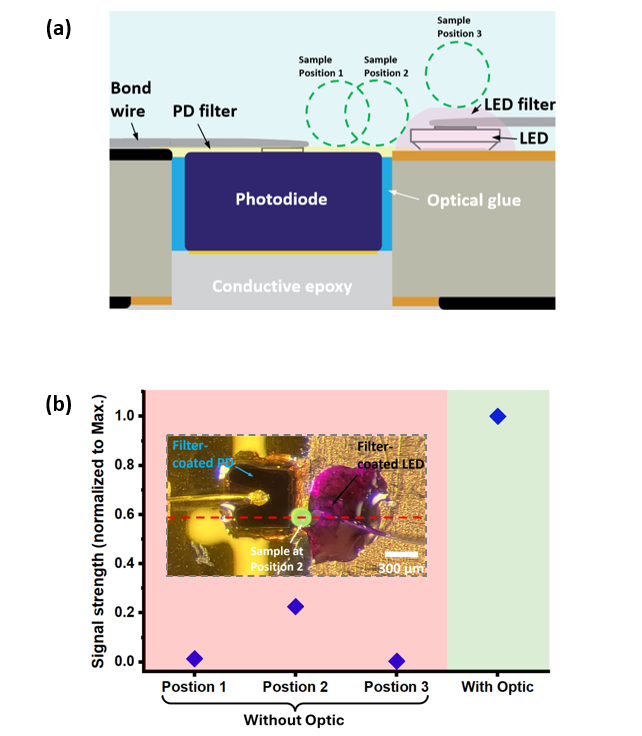


**Figure S10.** Comparison of signal strength between the IMOS and the simple configurations without the light guide: (a) Schematic illustration of three possible sample positions for configurations without the light guide. (b) measurement results across four different cases acquired using the acquisition system.


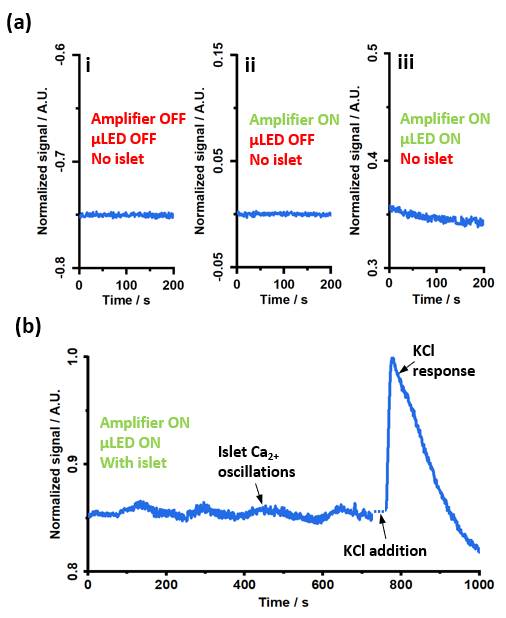


**Figure S11.** Evaluation of system background signals (normalized using the sum of background signals from the µPD amplification circuit and acquisition system as the baseline (signal = 0) and the peak islet response to KCl stimulation as the maximum (signal = 1), displayed on a consistent 20% scale of the full normalized range) without the warm-up process to allow a fair comparison with each experimental step. (a) Background signal measurements. (i) Background signals from the electrical acquisition system. (ii) Combined background signals from the acquisition system and µPD amplification circuit. (iii) Combined background signals from the acquisition system, µPD amplification circuit, and µLED operation (the LED was turned on abruptly). (d) Recorded signals in the presence of a pancreatic islet, showing Ca²⁺ oscillations and the islet’s response to KCl stimulation. All experimental results were acquired in a single recording to make a fair comparison.


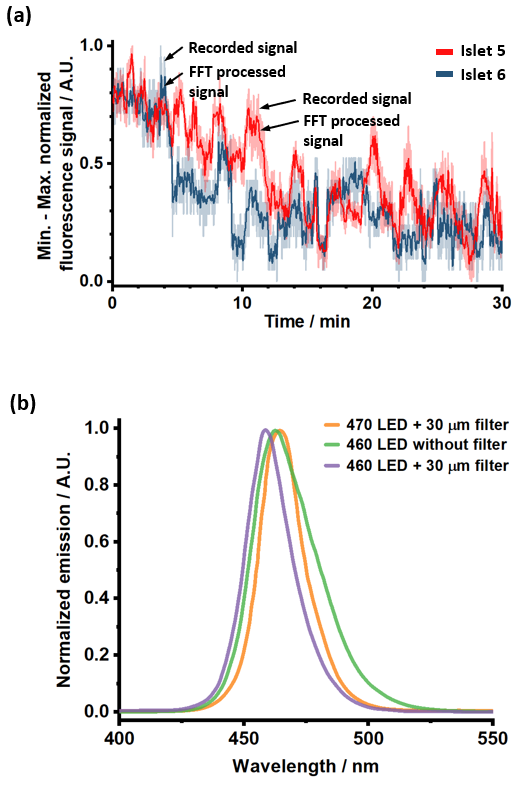


**Figure S12.** (a) 30-minute activities of two distinct islets (Islet 5 and Islet 6) recorded using IMOS with a filter-coated 470 nm peak emission µLED, processed using an FFT filter with a cutoff frequency of 0.16 Hz, and the results were normalized using Min.-Max. scaling (feature scaling) over the recording interval. (b) The comparison of emission spectra across three different cases: 470 nm peak emission µLED coated with a 30-µm thick absorptive optical filter (used for recordings shown in Figure 4f, Figure 5a, and Figure S12a), 460 nm peak emission without filter, µLED coated with the same type of 30-µm thick absorptive optical filter (used for recordings shown in Figure 5b, Figure 5c, and Figure S14).


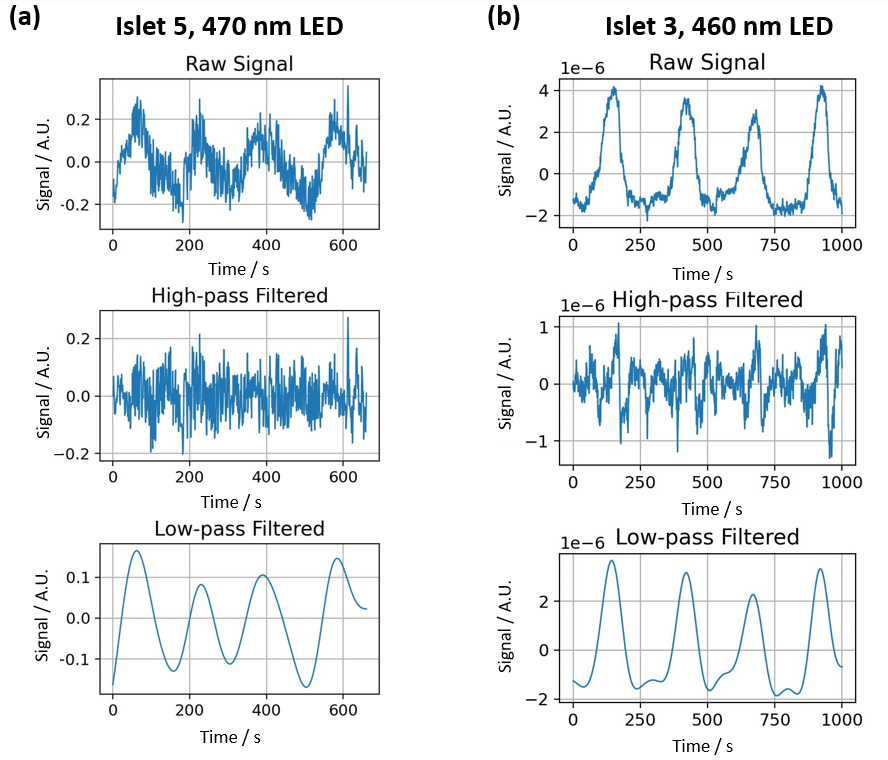


**Figure S13.** Estimation of the signal-to-noise ratio (SNR) for (a) 470 nm µLED configuration and (b) 460 nm µLED configuration. The raw signals were first processed using a high-pass filter with a cutoff frequency of 0.1 Hz, the processed signal was then used for calculating the background signal. Then we applied a low-pass filter with a cutoff of 0.01 Hz to separate the islet Ca²⁺ oscillation peaks. Finally, we estimated the SNR for both scenarios by taking the ratio between the average root-mean-square (RMS) amplitudes of Ca²⁺ oscillation peaks and background signals over the recording periods.


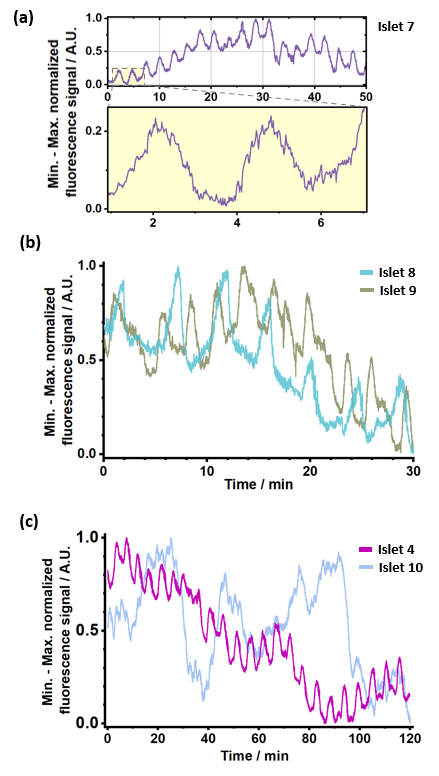


**Figure S14.** (a) 50-minute activity of an islet (Islet 7) recorded using IMOS with a filter-coated 460 nm peak emission µLED, the result was normalized using Min.-Max. scaling over the recording interval. (b) 30-minute activities of two distinct islets (Islet 8 and Islet 9) recorded using IMOS with a filter-coated 460 nm peak emission µLED, normalized using Min.-Max. scaling. (c) 2-hour activity of two distinct islets (Islet 4 and Islet 10) recorded using IMOS with a filter-coated 460 nm peak emission µLED, normalized using Min.-Max. scaling.
